# Closely Related *Vibrio alginolyticus* Strains Encode an Identical Repertoire of Caudovirales-Like Regions and Filamentous Phages

**DOI:** 10.3390/v12121359

**Published:** 2020-11-27

**Authors:** Cynthia Maria Chibani, Robert Hertel, Michael Hoppert, Heiko Liesegang, Carolin Charlotte Wendling

**Affiliations:** 1 Institut für Allgemeine Mikrobiologie, Christian-Albrechts-Universität zu Kiel, Am Botanischen Garten 1-9, 24118 Kiel, Germany; cchibani@ifam.uni-kiel.de; 2 Institute of Microbiology and Genetics, Department of Genomic and Applied Microbiology, Georg-August-University, 37077 Göttingen, Germany; hertel@b-tu.de (R.H.); hlieseg@gwdg.de (H.L.); 3 FG Synthetic Microbiology, Brandenburg University of Technology, 01968 Cottbus-Senftenberg, Germany; 4 Institute for Microbiology and Genetics, University of Göttingen, 37077 Göttingen, Germany; mhopper@gwdg.de; 5 GEOMAR, Helmholtz Centre for Ocean Research, Kiel, Düsternbrookerweg 20, 24105 Kiel, Germany; 6 ETH Zürich, Institute of Integrative Biology, Universitätstrasse 16, CHN D 33, 8092 Zürich, Switzerland

**Keywords:** vibriophages, filamentous phages, *Vibrio* virulence, prophages, zot, Inoviridae

## Abstract

Many filamentous vibriophages encode virulence genes that lead to the emergence of pathogenic bacteria. Most genomes of filamentous vibriophages characterized up until today were isolated from human pathogens. Despite genome-based predictions that environmental Vibrios also contain filamentous phages that contribute to bacterial virulence, empirical evidence is scarce. This study aimed to characterize the bacteriophages of a marine pathogen, *Vibrio alginolyticus* (Kiel-*alginolyticus* ecotype) and to determine their role in bacterial virulence. To do so, we sequenced the phage-containing supernatant of eight different *V. alginolyticus* strains, characterized the phages therein and performed infection experiments on juvenile pipefish to assess their contribution to bacterial virulence. We were able to identify two actively replicating filamentous phages. Unique to this study was that all eight bacteria of the Kiel-*alginolyticus* ecotype have identical bacteriophages, supporting our previously established theory of a clonal expansion of the Kiel-*alginolyticus* ecotype. We further found that in one of the two filamentous phages, two phage-morphogenesis proteins (Zot and Ace) share high sequence similarity with putative toxins encoded on the *Vibrio cholerae* phage CTXΦ. The coverage of this filamentous phage correlated positively with virulence (measured in controlled infection experiments on the eukaryotic host), suggesting that this phage contributes to bacterial virulence.

## 1. Introduction

Bacteriophages contribute significantly to bacterial adaptation and evolution. In particular, through bacterial lysis and subsequent killing, phages impose a strong selection pressure on their bacterial hosts. However, phages can also transfer genetic material to neighboring cells via horizontal gene transfer (HGT), thereby increasing bacterial genome plasticity [1] and ultimately bacterial fitness [2]. Temperate phages, including in particular filamentous phages, can become part of the bacterial genome either as chromosomally integrated prophages or as extra-chromosomal self-replicating elements [3]. They can further alter the bacterial phenotype through the introduction of accessory genes, a process known as lysogenic conversion [4].

One of the best-known examples of lysogenic conversion is the transformation of non-toxigenic *Vibrio cholerae* into deadly pathogens via the filamentous phage CTXΦ that carries the cholera toxin (CT) [4]. Since this first description of lysogenic conversion in 1996, several other filamentous phages, of which many carry bacterial virulence factors, have been discovered for the genus *Vibrio* as well as for other gram-negative bacteria [3]. For instance, two phages, VfO4K68 and VfO3K6, that carry the zona occludens toxin (Zot) and the accessory cholera enterotoxin (Ace), have been isolated from *Vibrio parahaemolyticus* [5]. Zot and Ace, which are essential for CTXΦ morphogenesis, are also hypothesized to display enterotoxin activity causing the classical diarrhea symptoms during cholera infections [6,7]. Zot and Ace are particularly common among filamentous vibriophages isolated from human pathogens, such as *V. cholerae* and *V. parahaemolyticus* [8,9,10] but are also predicted to be present in non-human pathogens such as *V. coralliilyticus* [11], and *V. anguillarum* [12,13] suggesting frequent HGT among different *Vibrio* species [14].

Other environmental vibrios that cause severe diseases to humans and marine animals include *V. splendidus, V. tubiashii* and *V. alginolyticus* [15,16,17,18]. While the virulence of these vibrio species is often attributed to multiple factors, such as temperature and host immunity [19], the contribution of filamentous phages and virulence factors encoded thereon is often not well characterized in environmental isolates. One reason might be that only fully-closed genomes are suitable to reliably identify integrated prophages. Another reason might be a research bias towards human pathogens. There are 330 closed *Vibrio* genomes (which have been deposited in the NCBI database, last accessed October 2020), of which more than 50% comprise human pathogens such as *V. cholerae* (89 genomes), *V. parahaemolyticus* (59 genomes) and *V. vulnificus* (25 genomes), while other environmental isolates are represented with significantly fewer strains. These include: *V. fluvialis* (11 genomes), *V. campbellii* (10 genomes), *V. anguillarum* (15 genomes), *V. alginolyticus* (28 genomes) and some others with fewer than 10 genomes per species. Additionally, to our knowledge only 18 filamentous vibriophages have so far been described in detail (NCBI, last accessed June 2020) of which 17 were isolated from human pathogens, namely, *V. cholerae* (11 phages) and *V. parahaemolyticus* (5 phages) and only one from an environmental fish pathogen *V. anguillarum*. 

The low number of well-described filamentous vibriophages is concerning and it is essential to start characterizing filamentous phages and their virulence factors in environmental isolates. A closer look at more than 1800 *Vibrio* genome sequences, covering 64 species revealed that 45% harbored filamentous phages that encoded Zot-like proteins [20]. Even though a detailed characterization of these phages and empirical evidence for their contribution to bacterial virulence is missing, this study suggests that filamentous vibriophages of non-human pathogens represent a massive reservoir of virulence factors [20]. In addition, filamentous vibriophages, which do not encode virulence factors, are still able to transfer them to other strains through specialized transduction [21,22]. Recombination between filamentous vibriophages can further result in hybrid phages, which are then able to vertically transmit toxins to other vibrios [21,22]. Indeed phage-mediated horizontal transfer of virulence genes seems to be a dynamic property among environmental vibrios [23]. Some filamentous vibriophages can infect distantly related species [24] and the host-range of some filamentous vibriophages increases in stressful environmental conditions that can be linked to global climate change [25].

The present study aimed to identify prophages and filamentous phages isolated from closely related *V. alginolyticus* strains, namely, the Kiel-*alginolyticus* ecotype [26], and to characterize their virulence potential. *V. alginolyticus*, a ubiquitous marine opportunistic pathogen can cause mass mortalities in shellfish, shrimp and fish, resulting in severe economic losses worldwide [27,28,29]. Additionally, wound infections and fatal septicemia in immunocompromised patients caused by *V. alginolyticus* have been reported in humans [30]. In contrast with the well-studied human pathogenic *Vibrio* species, there is limited information regarding phages from *V. alginolyticus* and their role in bacterial virulence. We thus performed controlled infection experiments on eukaryotic hosts, namely, juvenile pipefish, to be able to determine the virulence of this ecotype and to correlate it to the presence of a filamentous phage characterized in the present study.

## 2. Methods

### 2.1. Study Organisms

All eight *Vibrio alginolyticus* strains used in the present study (Table S1) were isolated either from the gut or the gills of six different pipefish (*Syngnathus typhle*) caught in the same sea-grass meadow in the Kiel Fjord in 2012 [31] and have been shown to cause mortality in juvenile pipefish ([26] and present study). Using a combination of PacBio and Illumina sequencing we generated eight complete genomes of these bacterial isolates in a previous study [26,32]. In brief, the genome assemblies were performed using the HGAP assembly software provided by PacBio [33]. To improve the quality of the genomes we mapped the Illumina data onto the HGAP assemblies, thereby correcting for sequencing errors. All sequenced genomes contained a ~3.47 Mbp chromosome 1 and a ~1.88 Mbp chromosome 2, with a %GC content of around 44% [32]. Based on a multi-scale comparative genomic approach we previously concluded that all eight bacteria belong to a unique ecotype, namely, the Kiel-*alginolyticus* ecotype, which evolved from a clonal expansion following niche adaptation [32]. To characterize the temperate phages of these eight *V. alginolyticus* strains, we now performed a comparative genomic analysis based on both the bacterial lysogen and the filamentous phage genomes sequenced from the supernatants.

### 2.2. Phage Isolation and Sequencing

#### 2.2.1. Prophage Induction

We isolated filamentous phages from all eight *V. alginolyticus* strains (Table S1) as described in [26]. In brief: bacteria were grown in liquid Medium101 (Medium101: 0.5% (w/v) peptone, 0.3% (w/v) meat extract, 3.0% (w/v) NaCl in MilliQ water) at 250 rpm and 25 °C overnight. Cultures were diluted 1:100 in fresh medium at a total volume of 20 mL and grown for another 2 h at 250 rpm and 25 °C to bring cultures into exponential growth before adding mitomycin C at a final concentration of 0.5 µg/mL. Afterwards, samples were incubated for 4 h at 25 °C and 230 rpm. After 4 h, samples were centrifuged at 2500× *g* for 5 min. The supernatant was sterile filtered using 0.45 µm pore size filter (Sarstedt, Nümbrecht, Germany). We added lysozyme from chicken egg white (10 µg/mL, SERVA Heidelberg, Germany) to disrupt the cell walls of potentially remaining host cells. RNAse A (Qiagen, Hilden, Germany) and DNAse I (Roche Diagnostics, Mannheim, Germany) were added each at a final concentration of 10 µg/mL to remove free nucleic acids as described in [26]. After incubation at 25 °C for 16 h phage particles were sedimented by ultracentrifugation using a Sorvall Ultracentrifuge OTD50B with a 60Ti rotor applying 200,000× *g* for 4 h. The supernatant was discarded and the pellet was dissolved in 200 µL TMK buffer (0.01 M Tris (pH 7.5), 0.005 M MgCl_2_ and 0.3 M KCl) and directly used for DNA isolation.

#### 2.2.2. Extraction of Phage DNA

DNA isolation was performed using a MasterPure DNA Purification kit from Epicenter (Madison, WI, USA). We added 200 µL 2x T&C-Lysis solution containing 1 µL proteinase K to the phage suspensions and centrifuged the samples for 10 min at 10,000× *g*. The supernatant was transferred to a new tube, mixed with 670 µL cold isopropanol and incubated for 10 min at −20 °C. DNA precipitation was performed by centrifugation for 10 min at 17,000× *g* and 4 °C. The DNA pellet was washed twice with 150 µL 75% ethanol, air-dried and re-suspended in DNase free water.

#### 2.2.3. Sequencing of Phage DNA

dsDNA for library construction was generated from viral ssDNA in a 50 µL reaction. The reaction was supplemented with 250 ng viral ss/DNA dissolved in water, 5 pmol random hexamer primer (#SO142, Thermo Scientific), 10 units Klenow Fragment (#EP0051, Thermo Scientific) and 5 µmol dNTPs each (#R0181, Thermo Scientific) and incubated at 37 °C for 2 h. The reaction was stopped by adding 1 µL of a 0.5 M EDTA pH 8 solution. The generated DNA was precipitated by adding 5 µL of a 3 M sodium acetate pH 5.2 and 50 µL 100% Isopropanol to the DNA solution, gently mixing and chilling for 20 min at −70 °C. DNA was pelleted via centrifugation at 17,000× *g*, 4 °C for 10 min. Pellets were washed twice with 70% ethanol. Remaining primers and viral ss/DNA were removed in a 50 µL reaction using 10 units S1 nuclease (#EN0321, Thermo Scientific) for 30 min at 25 °C. S1 nuclease was inactivated through the addition of 1µM 0.5 M EDTA pH 8 and incubation for 10 min at 70 °C. Consequently, ds/DNA was precipitated as described above and resolved in pure water. The presence of ds/DNA was verified via TAE gel electrophoresis in combination with an ethidium bromide staining and visualization via UV-light. NGS libraries were generated with the Nextera XT DNA Sample Preparation Kit (Illumina, San Diego, CA, USA), and the sequencing was performed on an Illumina GAII sequencer (Illumina, San Diego, CA, USA). All generated reads were checked for quality using the programs FastQC (Andrews 2010) and Trimmomatic [34].

### 2.3. Transmission Electron Microscopy

Transmission electron microscopy was carried out on a Jeol 1011 electron microscope (Eching, Germany) on the same samples that were used for whole-genome sequencing. Negative staining and transmission electron microscopy (TEM) of phage-containing particles was performed as described previously [35,36]. Phosphotungstic acid dissolved in pure water (3%; pH 7) served as staining solution.

### 2.4. Genomic Analysis

Annotation: We used the reference bacterial genomes [32] to annotate the sequenced filamentous phages. Initially, Prokka v1.11 [35] was used to generate an automated annotation. Prokka applies prodigal for gene calling [34], and was calibrated to the data set by using *Vibrio* as the genus reference (--genus *Vibrio* option) and an Inoviridae vibriophage protein database as a phage features reference database, which contained all available filamentous vibriophages described at the time of the analysis (Table S2). The initial annotation was manually curated using Interproscan and the database therein for every predicted CDS (https://www.ebi.ac.uk/interpro/search/sequence/), as well as hmmscan https://www.ebi.ac.uk/Tools/hmmer/search/hmmsearch) from HMMer. The naming of the genes from filamentous (pro)phages was done according to the review on filamentous phages by Mai-Prochnow et al. [3]. Finally we used viral zone to validate the proteins pI-pX predicted by both tools https://viralzone.expasy.org/113 and VIRFAM with the head-neck-tail module [37] to further classify the Caudovirales.

#### 2.4.1. Prediction of Prophage Regions

The bacterial genomes sequenced in [32] were scanned with PHASTER [38] to identify prophage like elements in each chromosome. Prophage quality and completion estimation was further supported by CheckV [39]. Predicted prophage regions were subsequently analyzed using Easyfig [40] for pairwise phage sequence comparisons and synteny comparisons with an *E*-value cut-off of 1 × 10^−10^. We used SnapGene Viewer (v.4.3.10) to generate a linear representation of all predicted prophage regions from each strain.

#### 2.4.2. Prediction of Active Replicating Phage Regions

The prediction of active phages was done by the identification and quantification of phage particle derived DNA that corresponds to predicted prophage regions. Trimmomatic [41] was used to trim the sequence adapter of the sequenced filamentous phage reads. All reads from filamentous phage DNA were mapped using bowtie2 [42] to the corresponding reference *V. alginolyticus* host genome. The generated mapping files were analyzed using TraV [43] to visualize filamentous phage DNA-derived coverage within the genomic context. Increased coverage (~10–1000× higher than the average coverage of the bacterial chromosome) was exclusively observed in genomic regions that have been identified by PHASTER [38] as intact phage regions. The increased coverage indicates an increased amount of DNA mapping on the prophage regions. This can be explained by prophages that have excised from the chromosome and entered a rolling circle replication mode. This was supported by the observation that cultures with increased coverage at those prophage regions generate a high amount of infective phage particles.

To estimate the relative phage production of each active phage locus we estimated the coverage of each locus relative to the coverage of the chromosome. Deeptools v.3.3.0 [44] was used to compute read coverage which was normalized using the reads per kilobase million (RPKM) method as follows: RPKM (per bin) = number of reads per bin/ (number of mapped reads (in millions) × bin length (kb)). The length of the bin used is 1 kb. As Illumina sequences were only available for seven of the eight *Vibrio* strains, we were not able to perform this analysis for strain K06K5.

#### 2.4.3. Comparative Genomic Analysis

We used the MUSCLE algorithm implemented in AliView v.1.15 [45] to conduct whole-genome alignments within all phage-groups that showed a high sequence similarity (68–100%) based on BLAST. Additionally, for prophages we performed alignments of the flanking host regions by comparing five genes located upstream and five genes located downstream of each prophage.

To investigate the phylogenetic relationship of *Vibrio* phage VALGΦ6 and *Vibrio* phage VALGΦ8 with other well-studied filamentous phages from different bacterial genera, we generated a phylogenetic tree using the VICTOR Virus Classification and Tree Building Online Resource [46] with all other filamentous vibriophages available at the time of analysis (Table S2). All pairwise comparisons of the nucleotide sequences were conducted using the Genome-BLAST Distance Phylogeny (GBDP) method [47] under settings recommended for prokaryotic viruses [46]. The resulting intergenomic distances were used to infer a balanced minimum evolution tree with branch support via FASTME including SPR postprocessing [48] for the formula D0. Branch support was inferred from 100 pseudo-bootstrap replicates each. Trees were rooted at the midpoint [49] and visualized with FigTree. Taxon boundaries at the species, genus and family level were estimated with the OPTSIL program [50], the recommended clustering thresholds [46] and an F-value (fraction of links required for cluster fusion) of 0.5 [51].

#### 2.4.4. Phage Genomes in Their Host Context

We compared the predicted prophage regions from the present study with potential prophage regions from all other so far published fully closed *V. alginolyticus* genomes (Table S4). To do so, we identified putative prophage regions on each chromosome using PHASTER [38] and compared those with the prophage regions from the present study using Easyfig [40]. The distribution of prophages across all 14 *V. alginolyticus* isolates was further visualized within the phylogenetic context of the host strains. For this, we generated a phylogenetic tree based on the core-genome alignment of 14 *V. alginolyticus* genomes (eight Kiel isolates and six non-Kiel isolates) [32]. This analysis was performed using the Bayesian Markov chain Monte Carlo (MCMC) method as implemented in MrBayes version 3.2.5 [52]. For further details see [32].

#### 2.4.5. Analysis of Virulence Factors

To determine potential virulence factors encoded on the two filamentous phages we determined the homology of the pI and the pVI proteins with pI and the pVI proteins associated with virulence in other vibriophages (Table S5) by means of protein alignments using AliView [45]. These two proteins, which are commonly referred to as Zot and Ace in pathogenic vibrios, are not only involved in phage morphogenesis but are also hypothesized to display enterotoxic activity causing the classical diarrhea symptoms during cholera infections [6].

#### 2.4.6. Data Deposit

GenBank files were deposited at NCBI for the two actively replicating filamentous phages VALGΦ6 (Accession number: MN719123) and VALGΦ8 (Accession number: MN690600).

### 2.5. Infection Experiments

We performed a controlled infection experiment to disentangle the role of the two filamentous phages in the virulence of the eight sequenced bacterial strains on juvenile pipefish as described in [26] with the following modification: Fish were infected via food. To do so, juvenile pipefish were kept together in one 2-L aquarium and fed with 10^3^ *Artemia* nauplii/mL which were previously exposed to a *V. alginolyticus* solution ~10^9^ CFU/mL (for 1 h) or seawater as control. We used three 2-L aquaria with 9–12 fish per *Vibrio* strain or control. Twenty-four hours post-infection, all fish were killed simultaneously with a lethal dose of MS222, which was directly added to the tanks. Subsequently, bacterial load was determined as colony forming units (CFU/mL). To avoid time-dependent bacterial growth, all dead fish were stored at +4 °C and the order of fish selected to quantify the CFU/mL was determined randomly. A detailed description of methods and the statistical analysis can be found in [26].

## 3. Results

### 3.1. General Overview

**Figure 1:**
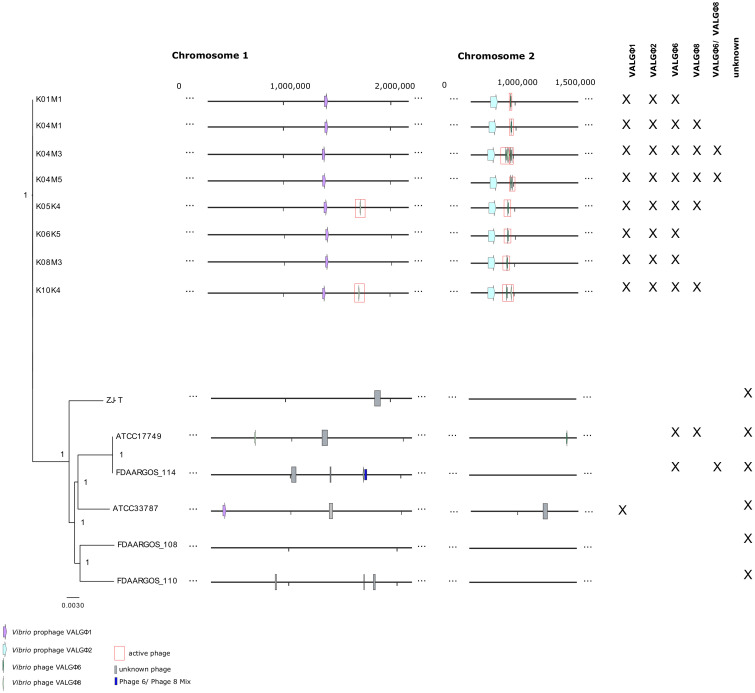
Left: Bayesian phylogenetic tree of all eight Kiel *V. alginolyticus* isolates (top) and six other fully sequenced *V. alginolyticus* isolates based on a core-genome alignment. Node-values represent bootstrap values; for additional information on the tree please see [26]. More information, regarding isolation source can be found in Tables S1 and S3. Middle: Whole chromosome alignment with prophage regions in colored boxes/arrows. Actively replicating prophages are marked in red. Blocks of the same color indicate prophage-types: purple, *Vibrio* prophage VALGΦ1; light blue, *Vibrio* prophage VALGΦ2; dark green, *Vibrio* phage VALGΦ6; light green, *Vibrio* phage VALGΦ8; dark blue, adjacent sequences of *Vibrio* phage VALGΦ6 and *Vibrio* phage VALGΦ8; grey, unknown phages. Right: presence (x) of selected vibriophages in each strain.

In previous studies [26,32] we sequenced the bacterial DNA of eight closely related *Vibrio alginolyticus* strains (Table S1). In the present study, we additionally sequenced the DNA extracted from the supernatant of mitomycin C treated liquid cultures of each strain. Within the eight sequenced *V. alginolyticus* genomes, we discovered two different prophage-like regions, each of which could be assigned to the order Caudovirales and two different prophage regions that could be assigned to the family Inoviridae (order Tubulavirales, Figure 1, Table S6). From the sequenced supernatant we could only identify filamentous phages but no Caudovirales. This suggests that in the tested *Vibrio* strains, filamentous phages are the only active replicating phages. To locate the exact positions in the bacterial chromosome of both filamentous phages, we performed a PHAGE-seq experiment [53]. To do so, we sequenced the bacterial cultures with and without prior exposure to mitomycin C. Both sequencing data sets revealed an increased coverage (estimated as abundance of reads) at Inoviridae loci relative to the coverage of the rest of the bacterial chromosome (Figure S1). Furthermore, mitomycin C treated and untreated cultures produced comparable amounts of phage particles. This supports our conclusion, that these strains produce a permanent amount of phage particle protected ssDNA which cannot be increased further by mitomycin C.

### 3.2. Caudovirales

Whole-genome comparison between the eight sequenced *V. alginolyticus* strains revealed the presence of two different prophage-like regions present in all eight strains: *Vibrio* prophage VALGΦ1 on chromosome 1 and *Vibrio* prophage VALGΦ2 on chromosome 2. Based on a classification of VALGΦ1 and VALGΦ2 encoded proteins using taxonomically assigned hidden Markov models as described in [54] both prophage-like regions were assigned as members of the Caudovirales family. None of these two prophages generated phage particles nor protein protected DNA in the experimental settings used in this study. Thus, a more thorough classification based on morphological characterization was not possible. Further, we did not find regions of increased coverage for these two prophage-like regions on the bacterial chromosomes (Figure S1). This indicates that both prophages were neither actively replicating in uninduced bacterial cultures nor able to switch to the lytic cycle upon induction with mitomycin C. We could not identify sequence similarities between both prophage-like regions, suggesting that they are genetically distinct. Both regions revealed specific genomes without significant similarities thereby indicating distinct viral types. However, all eight genomically distinct *V. alginolyticus* strains (Figure 1) revealed the presents of exactly these two prophage-like regions.

#### 3.2.1. *Vibrio* Prophage VALGΦ1

**Figure 2:**
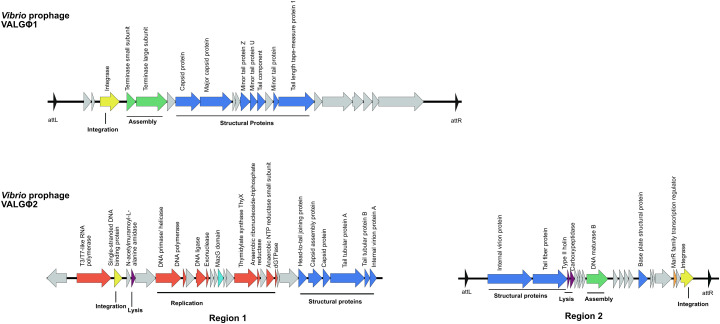
Synteny plots of *Vibrio* prophage VALGΦ1 (top), *Vibrio* prophage VALGΦ2 (bottom). ORFs are color-coded according to predicted function: red, replication; green, assembly; blue, structural proteins; yellow, integration; purple, lysis; orange, accessory genes; grey, hypothetical proteins.

*Vibrio* prophage VALGΦ1 has been assigned to Siphoviridae (type 1) as predicted by VIRFAM [37] and by ClassiPhage [54]. Its genome covers a 33.3 kbp long prophage-like region with a GC content of 46.06% and no tRNAs (Figure 2). According to PHASTER the total number of open reading frames (ORFs) is 22, with 10 ORFs assigned to one of five functional groups typical for phages (replication, assembly, structural proteins, integration, lysis) and 12 ORFs to hypothetical proteins. In contrast, CheckV assigned 13 CDS as viral genes. All ORFs were oriented in the same direction. Even though a high PHASTER score (ranging between 120 and 140) was assigned to this prophage-like region, which according to the software is indicative for intact prophages, we could not find phage particles corresponding to Caudovirales in induced and uninduced supernatants. This observation is further supported by a CheckV score of medium quality corresponding to 60.82% completeness. A closer look at the genome structure of VALGΦ1 reveals that this region is missing genes involved in replication and lysis. The lack of these genes might explain why we could not find any Caudovirales-like phage particles in our cultures.

*Vibrio* prophage VALGΦ1 is exclusively found on chromosome 1, where it has a unique integration site, which is identical across all eight sequenced bacterial strains. VALGΦ1 showed 100% sequence similarity across all eight sequenced lysogens. Likewise, the flanking regions on the bacterial chromosome (five genes upstream and five genes downstream of the integrated prophage) are identical across all sequenced clones. A BLAST search in NCBI GenBank using *Vibrio* prophage VALGΦ1 as query revealed the following two uncharacterized prophage regions as two closest hits: One region on the *V. diabolicus* strain FDAARGOS_105 integrated on chromosome 1 with a query cover of 77% and a similarity of 94.68% followed by an uncharacterized region on chromosome 1 of *V. alginolyticus* ATCC 33787 with a query cover of 57% and a similarity of 96.13%. These low query covers suggest that *Vibrio* prophage VALGΦ1 is a novel Caudovirales that is related to but distinct from other prophages found in chromosome 1 like replicons in the genus *Vibrio*.

#### 3.2.2. *Vibrio* Prophage VALGΦ2

The second Caudovirales-like region found in the present study, was initially identified as a complex of two prophage regions separated by a 2584 bp long gap. This prophage-like region has been assigned to Podoviridae (type 3) by VIRFAM [37]. According to predictions by PHASTER, the first part has a length of 26.3 kbp with a GC content of 49.37%, no tRNAs and a total of 29 ORFs, out of which 22 were assigned to one out of five functional phage-related groups and seven hypothetical proteins (Figure 2). These PHASTER predictions are in slight contrast to predictions by CheckV, which assigned four CDS as viral genes for all clones, except in the case of K04M5 where 8 CDS were assigned as viral genes. The second part, following the gap is 26.5 kbp long, with zero tRNAs, a GC content of 48.32% and comprises 20 OFRs, of which 12 could be assigned to phage-functional groups and eight as hypothetical proteins. The initial identification of two separate regions could lead to the interpretation of two different prophage-loci. However according to the gene composition of both regions (replication genes in region 1 and assembly genes in region 2) and the observation that there is no evidence that the sequence within the gap encodes some “non-phage” genes we think that this region comprises one prophage locus. *Vibrio* prophage VALGΦ2 has been assigned a low PHASTER score, ranging between 40 and 70 (which is indicative of a defective prophage according to the software), and its completeness was predicted to range between 35% (low quality) and 60% (medium quality) by CheckV. The results suggest that it does not contain all genes to be considered a functional prophage. This is further supported by our transmission electron microscopic investigation of the culture supernatant where we did not find any Caudovirales-like particles.

Limitation: The annotation of the VALGΦ1 and VALGΦ2 prophage-like regions is based on predictive tools. We obtained no phage read mappings obtained from particle protected DNA. Thus, there is no experimental support for the exact boundaries of the prophage regions. Additionally, CheckV is primarily developed to assess the quality of assembled viral genomes from metagenomes. However, in this case, the use of CheckV was performed on extracted regions of the predicted prophages and respective boundaries. The existence of a level of contamination due to the existence of host genes within the extracted prophage sequences further supports incorrect boundary predictions of prophage prediction tools.

### 3.3. Tubulavirales

#### 3.3.1. Phage Morphology

We determined the morphology of the phage particles from every strain using a transmission electron microscope (TEM, see Figure S2). According to the International Committee on Taxonomy of Viruses (ICTV), all phage particles were identified as filamentous phages.

#### 3.3.2. Phage Genomics

**Figure 3:**
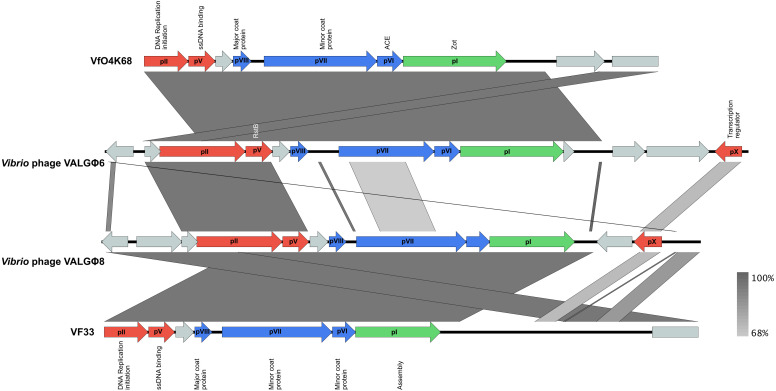
Synteny plots of *Vibrio* phage VALGΦ6 (second from top) and *Vibrio* phage VALGΦ8 (third from top) in comparison to VfO4K68 (top) and VF33 (bottom). ORFs are color-coded according to predicted function: red, replication; green, assembly; blue, structural proteins; grey, hypothetical proteins. Sequences with high sequence identity are indicated by dark grey and low identical sequences by light grey. pI-pX correspond to known filamentous phage proteins and putative homologues.

We could identify two different filamentous phages from the sequenced supernatant, namely, *Vibrio* phage VALGΦ6 and *Vibrio* phage VALGΦ8. These two filamentous phages were also predicted from the bacterial chromosomal sequences by PHASTER (PHASTER score between 120 and 150). However, the PHASTER predicted prophage boundaries contradicted with the region identified by the mapping of phage-particle derived DNA reads on the genome and we thus corrected them accordingly. Both filamentous phages contain single-stranded ssDNA genomes of 8.5 and 7.3 kb in size and a GC content of 44.6% and 46.3%, respectively. Both phages showed similar functional genes (typical for Inoviridae), which could be roughly grouped into three functional modules: Replication, assembly and structural proteins [3]. *Vibrio* phage VALGΦ6 and *Vibrio* phage VALGΦ8 share relatively little sequence similarity (50.72%), except for proteins involved in DNA replication (Figure 3).

*Vibrio* phage VALGΦ6 can be found exclusively on chromosome 2 in all eight strains, has a unique integration site and is identical across all eight bacterial strains. We found attL and attR sequences at the left and right end of the integrated prophage (TTAGCAACTTATAAA) suggesting that this phage inserts itself into the bacterial chromosome by site-specific recombination. However, genes corresponding to the well-described XerC/XerD system that results in site-specific integration of filamentous phages in *V. cholera* [55] have not been annotated by the genome annotation pipeline PROKKA. A more sensitive similarity-based search with BLAST and a domain-specific search using the HMMs from the Pfam database resulted in proteins with low similarity to XerC/XerD but not in a complete homologous operon. Thus, the mechanism of the site-specific integration remains to be discovered.

**Figure 4:**
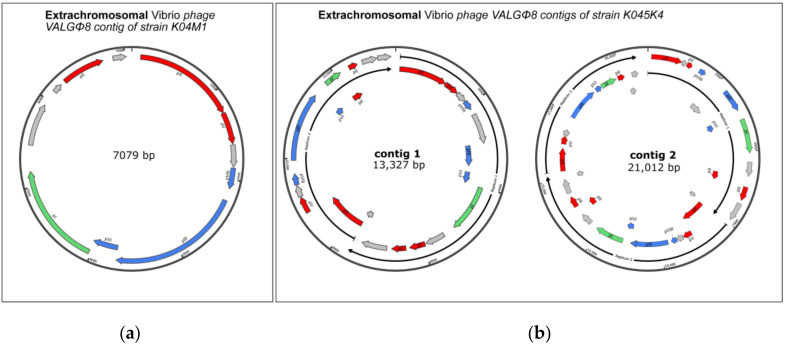
Extra-chromosomal contigs of *Vibrio* phage VALGΦ8 sequenced from (**a**) strain K04M1 and (**b**) strain K05K4 with the 2-replicon containing contig (left) and the 3-replicon containing contig (right). ORF-coding and protein names as in Figure 3.

*Vibrio* phage VALGΦ8 can only be found in five out of the eight bacterial strains and has two modes of inheritance: it can integrate on chromosome 2 (strain K04M3, K04M5, K10K4), chromosome 1 (Strain K05K4, K10K4) or exist extra-chromosomally (strain K04M1 without an intrachromosomal copy or in strain K05K4 with an intrachromosomal copy, Figure 4). When integrated on chromosome 2, *Vibrio* phage VALGΦ8 is always located directly behind *Vibrio* phage VALGΦ6 (Figure 1). In this case, the *Vibrio* phage VALGΦ6 specific attachment site flanks the entire phage locus, comprising *Vibrio* phage VALGΦ6 and *Vibrio* phage VALGΦ8. In strain K10K4, *Vibrio* phage VALGΦ8 is integrated on both chromosomes.

#### 3.3.3. Phage Activity and Within-Host Interaction

All loci predicted to correspond to filamentous phages represent actively replicating phages. First, we were able to detect filamentous phages in TEM pictures of all cultures (see Figure S2). Second, phage particles identified from induced and uninduced cultures exclusively contained DNA that matched at the filamentous phage loci (Figure S1, Table S3). Differences in coverage values of loci corresponding to filamentous phages suggest that the production of phage particles varies across phage regions and strains (Table S3). For those strains that did not contain *Vibrio* phage VALGΦ8, we evaluated the abundance of reads from uninduced cultures and found that all regions encoding *Vibrio* phage VALGΦ6 had increased coverage relative to the coverage of the chromosome. This indicates that in addition to the chromosomal copy of the phage genome actively replicating phages are present within the culture. However, in the presence of *Vibrio* phage VALGΦ8, the number of reads mapping on *Vibrio* phage VALGΦ6 encoding regions was reduced, but only when *Vibrio* phage VALGΦ8 was integrated on chromosome 2, and not when it existed exclusively extra-chromosomally or had an additional copy on chromosome 1. We thus conclude that depending on the genomic location of VALGΦ8, this phage has an impact on the replication of VALGΦ6.

#### 3.3.4. Phylogeny

**Figure 5:**
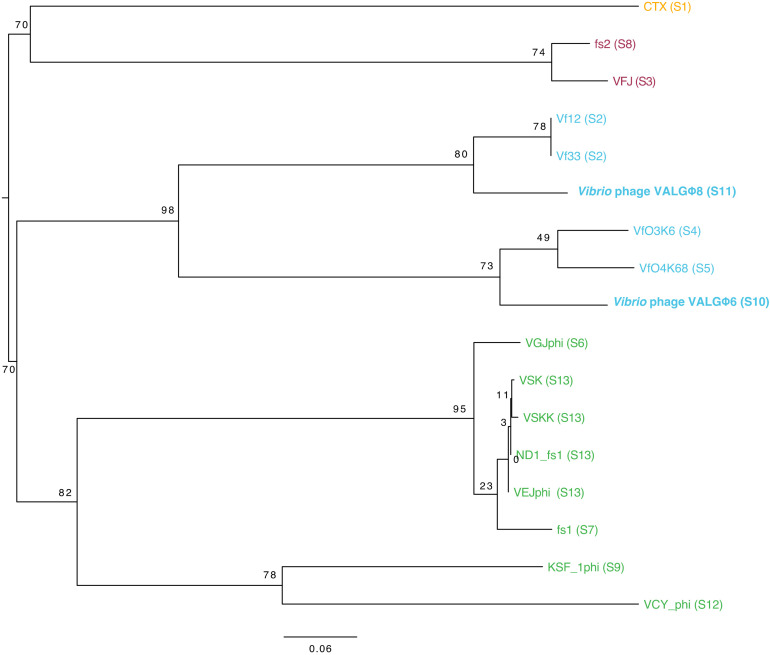
Mid-rooted phylogenomic GBDP tree inferred from VICTOR using the formula D0. The numbers above branches are GBDP pseudo-bootstrap support values from 100 replications. The OPTSIL clustering yielded twelve species clusters, four genus clusters and one cluster at the family level. Filamentous phages belonging to the same genus are color-coded. Same species are indicated by S1–S13 in brackets behind each phage name. Both filamentous phages, sequenced in the present study are highlighted with bold letters. Accession numbers of all filamentous phages can be found in Table S2.

Whole-genome alignment (Figure 3) and phylogenetic comparisons (Figure 5) suggest that *Vibrio* phage VALGΦ6 and *Vibrio* phage VALGΦ8 belong to the same genus but represent different viral species. *Vibrio* phage VALGΦ6 shares more sequence similarity with VfO4K68 and VfO3K6 (94.21% ANI), both isolated from *V. parahaemolyticus*. *Vibrio* phage VALGΦ8 shares more sequence similarity with VF33 and Vf12, also isolated from *V. parahaemolyticus* (94% and 93.98% ANI).

Previous comparative genomic analyses revealed that the eight *V. alginolyticus* strains isolated from the Kiel Fjord belong to a single ecotype, which forms a distinct cluster within the *V. alginolyticus* species [32]. To further investigate whether the phage-repertoire of the Kiel-*alginolyticus* ecotype is also distinct from other *V. alginolyticus* isolates we used PHASTER to predict prophages from all available closed non-Kiel *V. alginolyticus* genomes (six isolates in total) and found a total of 14 predicted prophage regions (Table S4). Comparisons between those uncharacterized vibriophages and prophages from the present study revealed a distinct distribution of vibriophages, which was nearly identical within the Kiel isolates, but very different among non-Kiel isolates. Furthermore, *Vibrio* phage VALGΦ6 and the *Vibrio* prophage VALGΦ2 are unique to the Kiel *alginolyticus* system. In contrast, we found integrated Inoviridae with high similarity (>97% ANI) to *Vibrio* phage VALGΦ8 in four of the six non-Kiel *V. alginolyticus* strains and one integrated Caudovirales that shared high sequence similarity (>97% ANI) with *Vibrio* prophage VALGΦ1 and had the same attL/attR sequence, in other words, CGTTATTGGCTAAGT (Figure 1, Figure S3). Despite having two, respectively one, unique integration site within the Kiel isolates, vibriophages from the non-Kiel isolates with high similarity to *Vibrio* phage VALGΦ8 and *Vibrio* prophage VALGΦ1 were mostly integrated on different positions in the respective chromosomes (Figure 1). All other uncharacterized prophages did not contain functional genes typical for Inoviridae suggesting that no other filamentous phage is present in the non-Kiel *V. alginolyticus* strains. In contrast to strains of the Kiel-*alginolyticus* ecotype, where most prophages were integrated on chromosome 2, only two out of the non-Kiel strains had prophages on chromosome 2 and one strain FDAARGOS_108 did not have a single prophage in its genome. Overall, the typical prophage composition consisting of *Vibrio* phage VALGΦ6 and *Vibrio* prophage VALGΦ2 on chromosome 2 together with *Vibrio* prophage VALGΦ1 on chromosome 1 is unique for our system and has not been found elsewhere. 

### 3.4. Virulence of Kiel V. alginolyticus Ecotypes

Comparative genomic analysis of the two phage morphogenesis proteins pI and pVI, revealed that only those of *Vibrio* phage VALGΦ6 share sequence similarity with pI and pVI of filamentous vibriophages associated with virulence where they are commonly referred to as virulence factors Zot and Ace (please see Table S5 for sequence similarity values). Sequence comparisons of Zot proteins encoded on 14 different vibrios revealed that the *Vibrio* phage VALGΦ6 encoded Zot is highly similar (>90% ANI) to Zot genes encoded on other closely related *Vibrio* species from the *harveyi* clade (such as *V. parahaemolyticus* or *V. campbellii,* Table S5).

**Figure 6:**
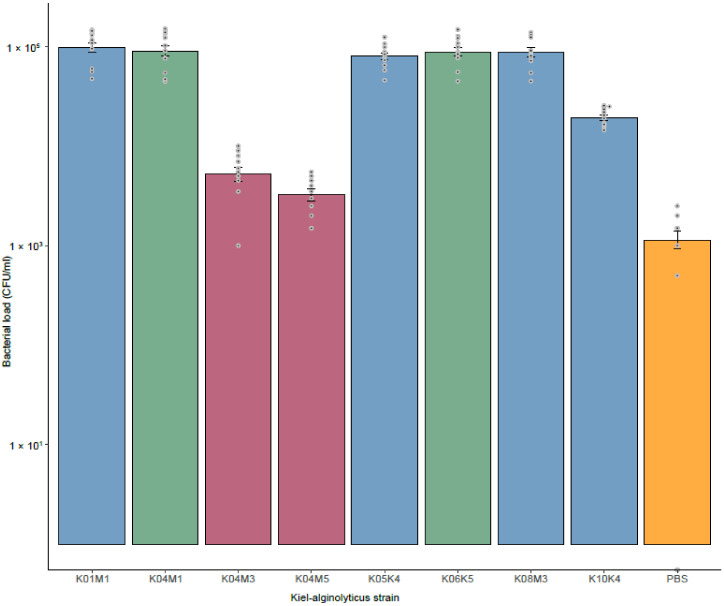
Bacterial load (CFU/mL) averaged over all pipefish per infected strain (mean ± s.e.) for each sequenced Kiel *V. alginolyticus* strain as well as PBS (orange). Strains which do not contain *Vibrio* phage VALGΦ8 are color coded in blue, strains which contain *Vibrio* phage VALGΦ8 but have a reduced coverage at the *Vibrio* phage VALGΦ6 locus are colored in bordeaux, and strains which contain *Vibrio* phage VALGΦ8 but do not have a reduced coverage at the *Vibrio* phage VALGΦ6 locus are colored in green.

Controlled infection experiments on juvenile pipefish revealed differences in total bacterial load among strains, which we take as a proxy for virulence. We found that fish infected with strains, that only encode *Vibrio* phage VALGΦ6 (i.e., K01M1, K06K5 and K08M3) had the highest infection load (Figure 6). In contrast, fish infected with strains with reduced coverage at the *Vibrio* phage VALGΦ6 locus (i.e., K04M5 and K04M3) had a similar infection load to fish from the control treatment. Thus, strains where *Vibrio* phage VALGΦ6 particle production is presumably reduced, cause a lower infection load and might be ultimately less virulent. This suggests that *Vibrio* phage VALGΦ6 contributes to virulence in *V. alginolyticus*.

## 4. Discussion

We present two novel filamentous vibriophages and two prophage-like regions isolated from eight different *Vibrio alginolyticus* strains all of which belong to the Kiel-*alginolyticus* ecotype. Using a combination of whole-genome sequencing, comparative genomic analyses and transmission electron microscopy we found two distinct prophage-like regions belonging to the order Caudovirales and two distinct chromosomal regions corresponding to actively replicating filamentous phages. Based on comparative genomic analyses we conclude that all four phage regions described in the present study have not been described before. Our main findings are that (1) closely related *V. alginolyticus* isolates, that were isolated from different eukaryotic hosts have identical bacteriophages; (2) filamentous phages from the present study have different modes of inheritance (intra-and extra-chromosomal) and might be able to suppress each other; and (3) horizontal gene transfer (HGT) of *Vibrio* phage VALGΦ6 containing the putative virulence factors Zot (zona occludens toxin) and Ace (accessory cholera enterotoxin) may contribute to the pathogenicity of the Kiel *V. alginolyticus* ecotype.

Closely related *V. alginolyticus* isolates, which were isolated from different eukaryotic hosts, have identical bacteriophages.

Three of the four described vibriophages in this study (i.e., *Vibrio* prophage VALGΦ1 on chromosome 1, *Vibrio* prophage VALGΦ2 and the filamentous *Vibrio* phage VALGΦ6 of chromosome 2) were present in all eight sequenced bacterial strains, had the same integration site and no variation in flanking regions on the chromosome (only exception: upstream region of *Vibrio* prophage VALGΦ2). Only the filamentous *Vibrio* phage VALGΦ8 was not present in all strains, displayed two different modes of inheritance (intra- and extra-chromosomal) and was able to integrate on both chromosomes. In a previous study we concluded that all eight bacterial strains belong to the Kiel-*alginolyticus* ecotype, which emerged after clonal expansion following HGT-driven niche adaptation [32]. The conserved positions found for *Vibrio* prophage VALGΦ1, *Vibrio* prophage VALGΦ2 and the filamentous *Vibrio* phage VALGΦ6 of chromosome 2 suggest that they were already present in the ancestor of the Kiel-*alginolyticus* ecotype. In contrast, *Vibrio* phage VALGΦ8 represents a new filamentous phage that is being acquired by some strains of the Kiel-*alginolyticus* ecotype through HGT.

The sampling design, spanning two different organs (gills or gut) from six different pipefish allows us to not only look at the vibriophages of closely related bacteria across eukaryotic hosts but also within eukaryotic hosts. We found more similarities within pipefish No. 4 (strains K04M1, K04M3 and K04M5) than across all six pipefish of which both contained *Vibrio* phage VALGΦ8. It is tempting to speculate that the high prevalence of *Vibrio* phage VALGΦ8 relative to all eight sequenced strains is a result of the proximity between strains inside the gut, which favors the rapid horizontal spread of *Vibrio* phage VALGΦ8. Future experiments would be needed to study the likelihood for *Vibrio* phage VALGΦ8 to establish successful infections and the circumstances which favor the different modes of inheritance (extra- or intra-chromosomal) and integration sites (chromosome 1 or chromosome 2).

### 4.1. Filamentous Phages Differ in Their Mode of Inheritance

While *Vibrio* phage VALGΦ6 was exclusively found at one integration site across all eight sequenced strains (exceptions: strains K04M3 and K04M5), *Vibrio* phage VALGΦ8 had different integration sites on both chromosomes and existed intra- and extra-chromosomally. We identified one, respectively two, extra-chromosomally closed circular contigs within the assembly of both *V. alginolyticus* strains K04M1 and K05K4 representing multimers of *Vibrio* phage VALGΦ8 (Figure 4). This indicates the presence of extra-chromosomal phage replicons in two out of the eight sequenced *V. alginolyticus* genomes. Filamentous phages typically multiply via the rolling circle replication (RCR) mechanism [3]. The strain K05K4 contains another copy of *Vibrio* phage VALGΦ8 integrated on chromosome 1 and the two extra-chromosomal contigs contain two, respectively three, copies of *Vibrio* phage VALGΦ8 (Figure 4). Considering that the contigs are derived from an assembly generated from long read data that contains reads that exceed the length of a single phage genome we hypothesize that the K05K4 extra-chromosomal contigs represent RCR intermediates of the integrated *Vibrio* phage VALGΦ8. However, to confirm or disprove this hypothesis’ experiments using knock-out versions of the intra-chromosomal copy of *Vibrio* phage VALGΦ8 in strain K05K4 have to be performed, which is beyond the scope of this study. In contrast, K04M1 does not contain an intrachromosomal version of *Vibrio* phage VALGΦ8 and the extra-chromosomal contig of K04M1 only consists of one phage replicon (Figure 4). This suggests that *Vibrio* phage VALGΦ8 is able to establish an extra-chromosomal infection without the need of an intrachromosomal copy.

### 4.2. Within-Host Competition Can Lead to the Reduction of Phage Producing Particles

In bacterial strains where both filamentous phages are present we observed a 10-fold reduced number of reads mapping at the *Vibrio* phage VALGΦ6 locus. We propose that the presence of *Vibrio* phage VALGΦ8, negatively affects the replication efficiency of *Vibrio* phage VALGΦ6. Even though, only two genes, namely, genes gII and gV, are involved in the phage DNA replication system, they have a high sequence similarity between both phages (Figure 3). Our data allow no conclusions on the involved mechanisms nor whether the highly similar gene products, pII and pV, do physically interact. This may be investigated by knock-out versions of strains containing both filamentous phages, as have been used in *B. licheniformis* DSM13 for Caudovirales phages [51], but has to remain unresolved until genetically accessible host strains for the phages become available. Thus, the results presented here are purely predictive based on read-mappings. Within-host competition is common among different Caudovirales prophages within the same host leading to strong selection for short lysis time [56]. To the best of our knowledge, nothing is known about within-host competition among filamentous phages and whether filamentous phages are able to suppress each other’s replication. However, studies on the classical biotype *V. cholerae*, where CTXΦ was present as an array of two truncated, fused prophages, found that even though the cholera toxin in expressed, no viral particles are produced [57]. Deficiencies in the array-structure and not mutations affecting individual CTXΦ genes have been suggested to be responsible for the absence of phage particle production. Similarly, we found the strongest reduction in coverage for *Vibrio* phage VALGΦ6 encoding regions in strain K04M3 and strain K04M5. Note, we did not find any genomic differences among the *Vibrio* phage VALGΦ6 regions in all eight bacterial strains. It is thus tempting to speculate that similar deficiencies in array structure are causing the coverage reduction of *Vibrio* phage VALGΦ6 encoding regions. Future studies unraveling within-host interactions of filamentous phages should elucidate whether such within-host competitions can influence the dynamics and evolutionary trajectories of filamentous phages.

### 4.3. HGT of Vibrio Phage VALGΦ6 Containing the Putative Virulence Factors Zot and Ace Might Have Led to the Emergence of the Pathogenic Kiel V. alginolyticus Ecotype

Filamentous phages are the most recognized vibriophages and present in almost every *Vibrio* genome sequenced to date for a detailed overview see [58]. Filamentous phages isolated from the Kiel-*alginolyticus* ecotype share more identity with filamentous phages isolated from *V. parahaemolyticus* than with other non-Kiel *V. alginolyticus* strains. This suggests a constant horizontal movement of filamentous phages between different *Vibrio* species without losing the ability to replicate in the old host(s). Indeed, some filamentous vibriophages have a very broad host range [24] and movement of vibriophages is not uncommon [59,60,61]. This will facilitate horizontal gene transfer (HGT) across species boundaries, which plays a significant role in the evolution of vibrios [58].

HGT also contributes substantially to the emergence of pathogenic vibrios from non-pathogenic environmental populations [58]. For instance, CTXΦ is able to transduce the cholera toxin (CT) from *V. cholera* to *V. mimicus* leading to the emergence of a pathogenic *V. mimicus* strain [59,60]. Many vibriophages contain virulence genes responsible for severe gastro-intestinal diseases [62,63]. For instance, almost 80% of clinical *V. parahaemolyticus* strains contain filamentous phages, encoding the zona occludens toxin (Zot) [20]. Furthermore, non-human pathogens, such as *V. coralliilyticus* and *V. anguillarum* contain prophage-like elements encoding Zot, suggesting frequent horizontal gene transfer (HGT) of Zot via prophages among vibrios [14]. In the present study, we found one filamentous phage, namely, *Vibrio* phage VALGΦ6, that contains the putative virulence factors Ace and Zot, which are common among vibrios [64]. Considering the high sequence identity between *Vibrio* phage VALGΦ6 and phages isolated from *V. parahaemolyticus* this might represent another example where the movement of a filamentous phage across species boundaries leads to the transfer of virulence factors possibly being responsible for the pathogenicity of Kiel *V. alginolyticus* ecotypes. Controlled infection experiments revealed a close link between virulence and coverage of the region encoding for *Vibrio* phage VALGΦ6. Strains, for which we observed a strong reduction in sequencing coverage at the region encoding for *Vibrio* phage VALGΦ6, caused a reduced infection load compared to strains with a high coverage at this locus. This suggests that the low coverage may result in a reduced number of viral particles and potentially a reduced production of both toxins, which may ultimately result in lower virulence. However, to ultimately prove that Ace and Zot encoded on *Vibrio* phage VALGΦ6 are causing the virulence of our isolates we would need a strain that does not contain *Vibrio* phage VALGΦ6 for controlled infection experiments.

## 5. Conclusions

By characterizing two novel filamentous vibriophages isolated from environmental strains we increase the current knowledge on filamentous vibriophages, which is as of October 2020 heavily biased towards human pathogens. We also show that non-human pathogenic vibrios represent a reservoir of filamentous phages, which can contain virulence factors and potentially move between species leading to the emergence of pathogens. We want to encourage future studies on the phage-repertoire and the virulence factors of other non-human pathogenic vibrios. By looking at a wider range of *Vibrio* species we will then considerably expand our knowledge on the types of mobile genetic elements in *Vibrio* and in particular how they influence the virulence and evolution of this species.

## Supplementary Materials

The following are available online at https://www.mdpi.com/1999-4915/12/12/1359/s1, Figure S1: Coverage (y-axis) for chromosome 1 (left) and chromosome 2 (right) for seven sequenced strains, Figure S2: Electron micrographs of filamentous phages of V. alginolyticus K10K4 ) and V. alginolyticus K01M1, Figure S3: Whole genome alignment of predicted prophage regions from non-Kiel V. alginolyticus strains and phages identified in the present study Table S1: *Vibrio alginolyticus* genomes sequenced in the present study, Table S2: Filamentous vibriophages used as references for the annotation of the Kiel *alginolyticus* phages in the present study, Table S3: All prophage regions predicted by PHASTER for each chromosome and the coverage* of Illumina reads generated from phage particles relative to the coverage of the entire chromosome, Table S4: Prophage regions found in all available closed non-Kiel *alginolyticus* strains predicted by PHASTER and their similarity to Kiel *alginolyticus* phages, Table S5: Sequence similarity (%) between the two phage morphogenesis proteins and potential virulence factors pI (Zot) and pVI (ACE) encoded on *Vibrio* phage *VALG*Φ6 and those found in prophage regions of other Vibrio species, Table S6: All prophages and filamentous phages and their characteristics determined for all eight Vibrio alginolyticus strains isolated from different pipefish in the Kiel Fjord.

## Abbreviations

HGT, horizontal gene transfer; MGE, mobile genetic element; CT, cholera toxin; Zot, zona occludens toxin; Ace, accessory cholera enterotoxin; TEM, transmission electron microscopy; MCMC, Markov chain Monte Carlo.
