# Supplementary material for: Closely Related Vibrio alginolyticus Strains Encode an Identical Repertoire of Caudovirales-Like Regions and Filamentous Phages
Source: Viruses. 2020 Nov 27;12(12):1359. doi: 10.3390/v12121359 (PMC7761403; doi:10.3390/v12121359)
Supplement: Supplementary file 1 [file viruses-12-01359-s001.pdf]

viruses

MDPI

Closely related *Vibrio alginolyticus* strains encode an identical repertoire of Caudovirales and Tubulavirales

Cynthia Maria Chibani, Robert Hertel, Michael Hoppert, Heiko Liesegang and Carolin C. Wendling

Supplementary material

Table S1. *Vibrio alginolyticus* genomes sequenced in the present study. Shown are the different organs and pipefish of isolation, the presence of *Inoviridae*, and the accession number of both chromosomes and if available of extrachromosomal phage replicons.

Strain	Pipefish	Organ	Inoviridae	Accession Number (Chromosome I/II)	Extrachromosomal Phages
K01M1	1	Gut	VALGΦ6	CP017889.1/ CP017890.1	
K04M1	4	Gut	VALGΦ6, VALGΦ8	CP017891.1/ CP017892.1	CP017895.1
K04M3	4	Gut	VALGΦ6, VALGΦ8	CP017896.1/ CP017897.1	
K04M5	4	Gut	VALGΦ6, VALGΦ8	CP017899.1/ CP017900.1	
K05K4	5	Gills	VALGΦ6, VALGΦ8	CP017902.1/ CP017903.1	CP017905.1/ CP017906.1
K06K5	6	Gills	VALGΦ6	CP017907.1/ CP017908.1	
K08M3	8	Gut	VALGΦ6	CP017913.1/ CP017914.1	
K10K4	10	Gills	VALGΦ6, VALGΦ8	CP017911.1/ CP017911.1	

Table S2. Filamentous vibriophages used as references for the annotation of the Kiel *alginolyticus* phages in the present study. Note, at the time of analysis this set represented all available filamentous vibriophages.

Vibrio Phage	Accession number	Size (bp)	Host
Vibrio phage fs1	NC_004306.1	6,340	Vibrio cholerae O139
Vibrio phage fs2	NC_001956.1	8,651	Vibrio cholerae O139
Vibrio phage VfO4K68	NC_002363.1	6,891	Vibrio parahaemolyticus
Vibrio phage VEJphi	NC_012757.1	6,842	Vibrio cholerae
Vibrio phage VFJ	NC_021562.1	8,555	*Vibrio cholerae* O139 serogroup strain ICDC-4470
Vibrio phage KSF-1phi	NC_006294.1	7,107	Vibrio cholerae
Vibrio phage VGJphi	NC_004736.1	7,542	Vibrio cholerae
Vibrio phage VSK	AF453500.3	6,882	Vibrio cholerae O139
Vibrio phage VCY-phi	NC_016162.1	7,103	*Vibrio cholerae* str. 10E09PW02
Vibrio phage Vf33	NC_005948.1	7,965	Vibrio parahaemolyticus
Vibrio phage VfO3K6	NC_002362.1	8,784	Vibrio parahaemolyticus
Bacteriophage VSKK	AF452449.2	6,834	*Vibrio cholerae* O139 strain BO4
Vibrio phage ND1-fs1	AB572858.1	6,856	*Vibrio cholerae* O139 strain AI4450
Vibrio phage Vf12	AB012574.1	7,965	Vibrio parahaemolyticus
Vibrio phage CTX	HQ224500.1	10,638	Vibrio cholerae KMN002

Table S3. All prophage regions predicted by PHASTER for each chromosome and the coverage* of Illumina reads generated from phage particles relative to the coverage of the entire chromosome. NA coverage for strain K06K5 is because of missing Illumina sequences for that strain. Chr = Chromosome. PHASTER scores for VALGΦ1 were between 120-140, for VALGΦ2 40-90, and Tubulavirales between 120-150.

Strain	Chr.	Phage type	Position	Relative coverage [RPKM]
		**Caudovirales**		
K01M1	1	Vibrio prophage VALGΦ1	1386230 - 1416561	0
K04M1	1	Vibrio prophage VALGΦ1	1389760 - 1420091	0
K04M3	1	Vibrio prophage VALGΦ1	1367485 - 1397816	0
K04M5	1	Vibrio prophage VALGΦ1	1367485-1397816	0
K05K4	1	Vibrio prophage VALGΦ1	1383927-1414258	0
K06K5	1	Vibrio prophage VALGΦ1	1389236-1419567	0
K08M3	1	Vibrio prophage VALGΦ1	1384771-1415102	0
K10K4	1	Vibrio prophage VALGΦ1	1367484-1397815	0
K01M1	2	Vibrio prophage VALGΦ2	823633 - 849961	0
			852546 - 879104	
K04M1	2	Vibrio prophage VALGΦ2	802745 - 829073	0
			831658 - 858216	
K04M3	2	Vibrio prophage VALGΦ2	802746 - 829074	0
			826940 - 853486	
K04M5	2	Vibrio prophage VALGΦ2	832920 - 859248	0
			857114 - 883660	
K05K4	2	Vibrio prophage VALGΦ2	815441 - 841769	0
			844354 - 870912	
K06K5	2	Vibrio prophage VALGΦ2	802739 - 829067	0
			831652 - 858210	
K08M3	2	Vibrio prophage VALGΦ2	802751 - 829079	0
			831664 - 858222	
K10K4	2	Vibrio prophage VALGΦ2	802733 - 829061	0
			831646 - 858204	
		**Tubulavirales**		
K01M1	2	Vibrio phage VALGΦ6	966016 - 974545	124027
K04M1	2	Vibrio phage VALGΦ6	945128 - 953658	164987
K04M3	2	Vibrio phage VALGΦ6	945129 - 953754	30694
K04M3	2	Vibrio phage VALGΦ6	979135 - 986319	7042
K04M5	2	Vibrio phage VALGΦ6	980896 - 983928	623
K05K4	2	Vibrio phage VALGΦ6	957739 - 965822	108737
K06K5	2	Vibrio phage VALGΦ6	945122 - 953651	NA
K08M3	2	Vibrio phage VALGΦ6	945134 - 953663	309280
K10K4	2	Vibrio phage VALGΦ6	945116 - 953645	12620
K04M3	2	Vibrio phage VALGΦ8	954112 - 961313	93647
K04M3	2	Vibrio phage VALGΦ8	961303 - 968746	122513
K04M3	2	Vibrio phage VALGΦ8	988262 - 993332	71539
K04M5	2	Vibrio phage VALGΦ8	984286 - 990120	745243
K05K4	1	Vibrio phage VALGΦ8	1718233 -1725757	509234
K10K4	1	Vibrio phage VALGΦ8	1701790-1709101	338893
K10K4	2	Vibrio phage VALGΦ8	977819 - 984038	432296

* Coverage values are normalized per region and scaled per million. Unit is RPKM = reads per kilobase million

Table S4. Prophage regions found in all available closed non-Kiel *alginolyticus* strains predicted by PHASTER and their similarity to Kiel *alginolyticus* phages (No prophage region was predicted for strain FDAARGOS_108).

Strain and Isolation	Acc. Number	Chr.	Region	Position	Nr. Proteins	GC	kbp	completeness	Kiel similarity
ATCC17749	CP006718.1	1	Region1	670315-679272	15	44.03	8.9	intact	VALGΦ8
Spoiled horse mackerel			Region2	1274126-1321950	42	43.78	47.8	intact	
Japan	CP006719.1	2	Region1	1326210-1334787	12	44.75	8.5	intact	VALGΦ8
ATCC33787	CP013484.1	1	Region1	386162-416625	20	45.91	30.4	intact	VALGΦ1
Seawater			Region2	1349875-1379647	38	29.7	29.7	intact	
Oahu, Hawai	CP013485.1	2	Region1	1182463-1211012	40	46.56	28.5	questionable	
FDAARGOS_110	CP014040.1	1	Region1	880871-890638	8	45.77	9.7	defect	
unknown			Region2	1699837-1704038	11	41.98	41.98	defect	
			Region3	1786647-1802662	18	18	42.64	defect	
FDAARGOS_114	CP014045.1	1	Region1	1015704-1052063	43	43.26	36.3	intact	
unknown			Region2	1363248-1370095	13	44.19	6.8	defect	
			Region3	1659847-1668804	14	44.01	8.9	intact	VALGΦ8
			Region4	1674237-1695783	36	44.33	21.4	intact	VALGΦ8/ VALGΦ6 mix
ZJ-T	CP016224.1	1	Region1	1851721-1904717	46	46.06	52.9	intact	
diseased *Epinephelus coioides*									
Zhanjiang, China									

Table S5. Sequence similarity (%) between the two phage morphogenesis proteins and potential virulence factors pI (Zot) and pVI (ACE) encoded on *Vibrio* phage *VALGΦ6* and those found in prophage regions of other Vibrio species.

Protein	Vibrio host	Accession Number	Sequence Similarity (%)
Zot	*V. cholerae*	AAL09684.1	24.56
Zot	*V. cholerae*	ACV73683.1	24.34
Zot	*V. parahaemolyticus*	KZW05498.1	99.78
Zot	*V. parahaemolyticus*	WP_025818349.1	99.35
Zot	*V. neocaledonicus*	WP_137282009.1	99.78
Zot	*V. diabolicus*	WP_104972604.1	98.92
Zot	*V. harveyi*	WP_005441625.1	90.02
Zot	*V. jasicida*	WP_104047307.1	89.59
Zot	*V. owensii*	WP_039985951.1	90.67
Zot	*V. campbellii*	WP_050905068.1	85.47
Zot	*V. alginolyticus*	WP_047101102.1	99.57
Zot	*V. alginolyticus*	WP_138940288.1	99.13
Zot	*V. alginolyticus*	WP_053303055.1	99.13
Ace	*V. cholerae*	UP000000584	21.88
Ace	*V. parahaemolyticus*	WP_069547465.1	100
Ace	*V. sp. JCM 18905*	GAJ76692.1	100
Ace	*V. sp. JCM 18904*	GAJ69591.1	99.02

Table S6. All prophages and filamentous phages and their characteristics determined for all eight Vibrio alginolyticus strains isolated from different pipefish in the Kiel Fjord.

Prophage	Order	length [kbp]	GC-content [%]	Phaster prediction
Vibrio prophage VALGΦ1	Caudovirales	33.3	46.06	intact
Vibrio prophage VALGΦ2	Caudovirales	26.4 [2584bp] 26.5	49.37 and 48.32	questionable
Vibrio phage VALGΦ6	Tubulavirales	8.5	44.6	intact
Vibrio phage VALGΦ8	Tubulavirales	7.3	46.3	intact

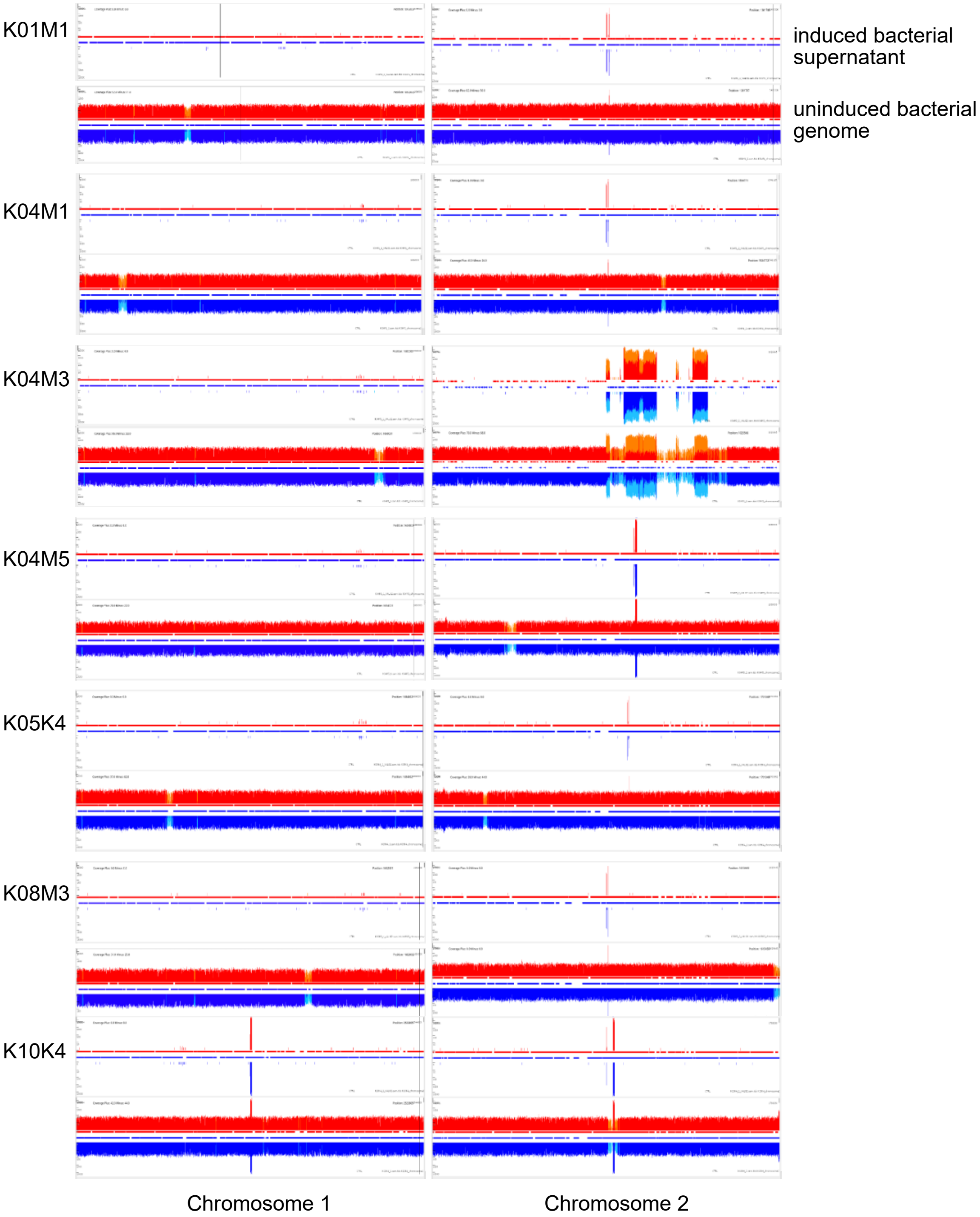

Figure S1. Coverage (y-axis) for chromosome 1 (left) and chromosome 2 (right) for seven sequenced strains (note: strain K06K5 is missing due to missing Illumina sequences). Each strain is represented by two images: Top: induced supernatant, bottom: uninduced whole genome sequence of bacteria. Regions of increased coverage correspond to active regions of filamentous phages. Regions of increased coverage in uninduced supernatant are identical with regions of increased coverage in bacterial genome indicating that induced and uninduced cultures produce comparable amounts of filamentous phages. Blue: negative strand, red: positive strand, light-bkue and orange represent multimappings.

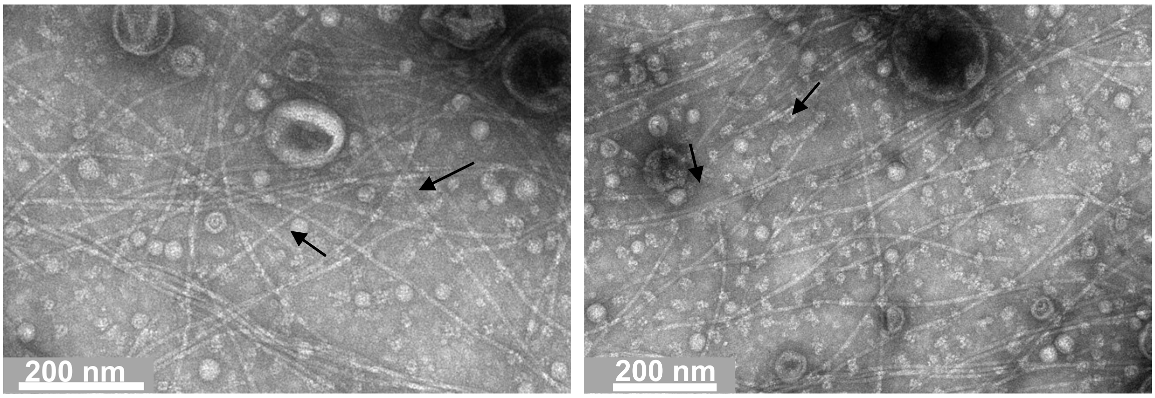

Figure S2. Electron micrographs of filamentous phages of V. alginolyticus K10K4 (Mixture Vibrio phage VALGΦ6 and Vibrio phage VALGΦ8) and V. alginolyticus K01M1 (Vibrio phage VALGΦ6). Arrows point to single filaments.

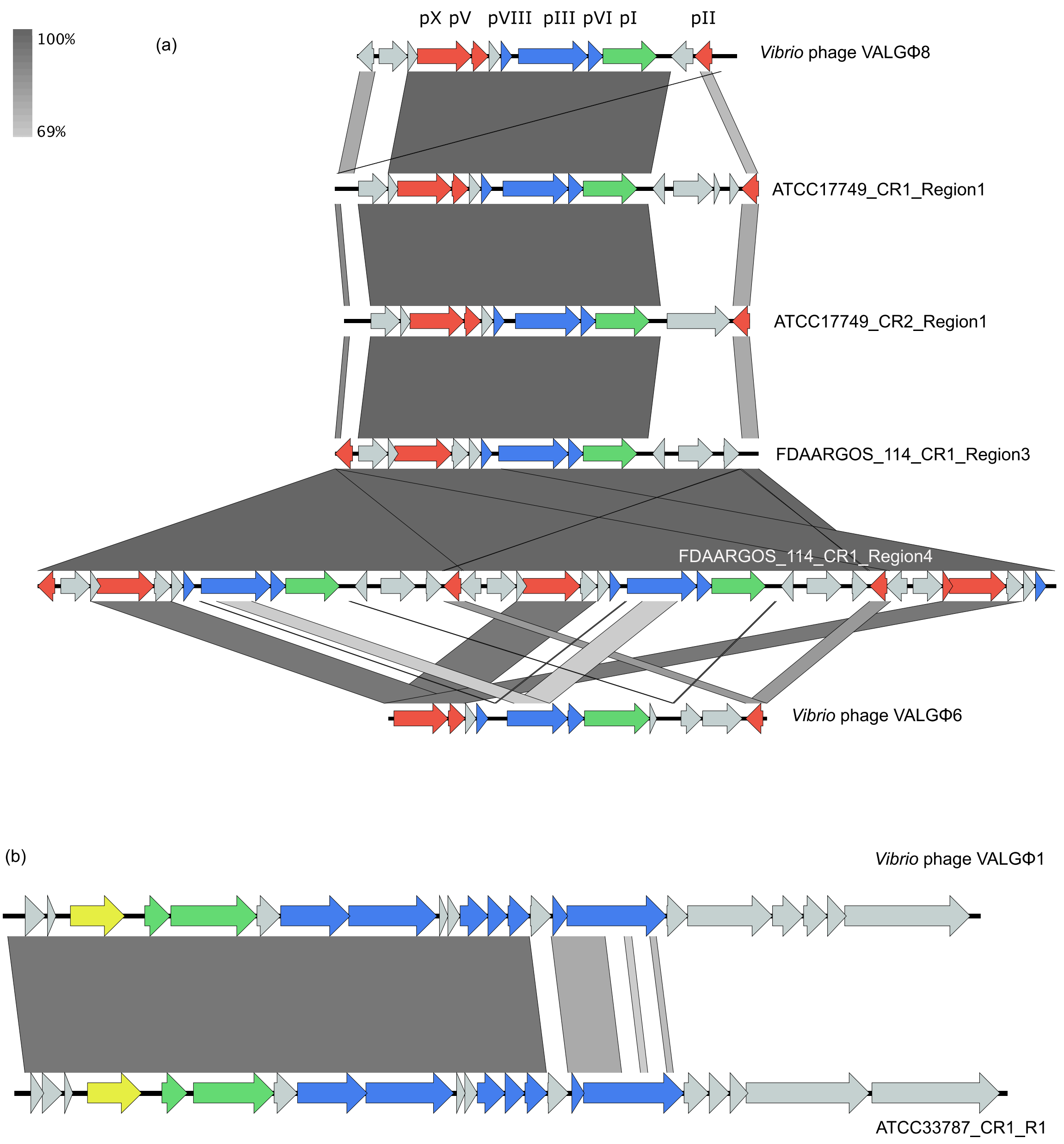

Figure S3. Whole genome alignment of predicted prophage regions from non-Kiel V. alginolyticus strains and phages identified in the present study. (a) Predicted prophage regions that show similarity with Vibrio phage VALGΦ8 and Vibrio phage VALGΦ6, ORFs are color-coded according to predicted function for Inoviridae: red: replication, green: assembly, blue: structural proteins, grey: hypothetical proteins. pI – pX correspond to known filamentous phage proteins and putative homologues. (b) Region with similarity to Vibrio phage VALGΦ1. ORFs are color-coded according to predicted function: green: assembly, blue: structural proteins, yellow: integration, grey: hypothetical proteins. High homologous sequences are indicated by dark grey and low homologous sequences by light grey.
